# COVID-19 and VILI: developing a mobile app for measurement of mechanical power at a glance

**DOI:** 10.1186/s40635-021-00372-0

**Published:** 2021-02-09

**Authors:** Angelo Senzi, Marco Bindi, Iacopo Cappellini, Lucia Zamidei, Guglielmo Consales

**Affiliations:** 1 Department of Anaesthesia and Intensive Care, Santo Stefano Hospital, Prato, Italy; 2 Department of Information Engineering, University of Florence, Florence, Italy; 3 Department of Anaesthesia and Intensive Care, Santo Stefano Hospital, Prato, Italy

**Keywords:** Mechanical power, COVID-19, App, Mobile application, VILI, Mathematical computation

## Abstract

The COVID-19 pandemic has increased the need for a bedside tool for lung mechanics assessment and ventilator-induced lung injury (VILI) monitoring. Mechanical power is a unifying concept including all the components which can possibly cause VILI (volume, pressures, flow, respiratory rate), but the complexity of its mathematical computation makes it not so feasible in routine practice and limits its clinical use. In this letter, we describe the development of a mobile application that allows to simply measure power associated with mechanical ventilation, identifying each component (respiratory rate, resistance, driving pressure, PEEP volume) as well. The major advantage, according to the authors who developed this mathematical description of mechanical power, is that it enables the quantification of the relative contribution of its different components (tidal volume, driving pressure, respiratory rate, resistance). Considering the potential role of medical apps to improve work efficiency, we developed an open source Progressive Web Application (PWA), named “PowerApp” (freely available at https://mechpower.goodbarber.app), in order to easily obtain a bedside measurement of mechanical power and its components. It also allows to predict how the modification of ventilatory settings or physiological conditions would affect power and each relative component. The "PowerApp" allows to measure mechanical power at a glance during mechanical ventilation, without complex mathematical computation, and making mechanical power equation useful and feasible for everyday clinical practice.

## To the Editor,

The COVID-19 pandemic has increased the need for a bedside tool for lung mechanics assessment and ventilator-induced lung injury (VILI) monitoring.

As described by Gattinoni [1], mechanical power is a unifying concept including all the components which could cause VILI (volume, pressures, flow, respiratory rate). Attempting to define a safe threshold, Guerin found that mechanical power of respiratory system above 12 J/min was associated with reduced survival [2].

In a recent retrospective analysis of ARDS paralyzed and mechanically ventilated patients, power normalized to the compliance (or to the amount of well-aerated tissue) is independently associated to the intensive care mortality [3].

Mechanical power formulation requires the patient to be passively ventilated and enables the quantification of the relative contribution of its different components (tidal volume, driving pressure, respiratory rate, resistance) predicting the effects of their changes during ventilator setting [1], nevertheless its highly complex computation makes its use unfeasible in routine practice. For this reason, Chiumello et al. proposed a simple surrogate equation for volume-controlled ventilation, but it carries a small bias (overestimation) [4].

Considering the wide availability of the Internet and the potential role of medical apps to improve work efficiency, we developed a Progressive Web Application (PWA) named “PowerApp”, in order to easily obtain a bedside measurement of mechanical power and its components in mechanically ventilated patients. The effects of each component on MP are not always easily predictable in clinical practice, because changing one parameter will often modify others [5].

Entering data available from the ventilator in the “PowerApp” allows clinicians not only to promptly calculate the mechanical power (partitioned in its elastic, resistive and PEEP component), but also to predict how the modification of ventilator settings or physiological conditions might affect power and each relative component. Currently, the app can be shared via a URL link (freely available at https://mechpower.goodbarber.app) or can be found via a web search engine, allowing for wide sharing of the content.

**Fig. 1:**
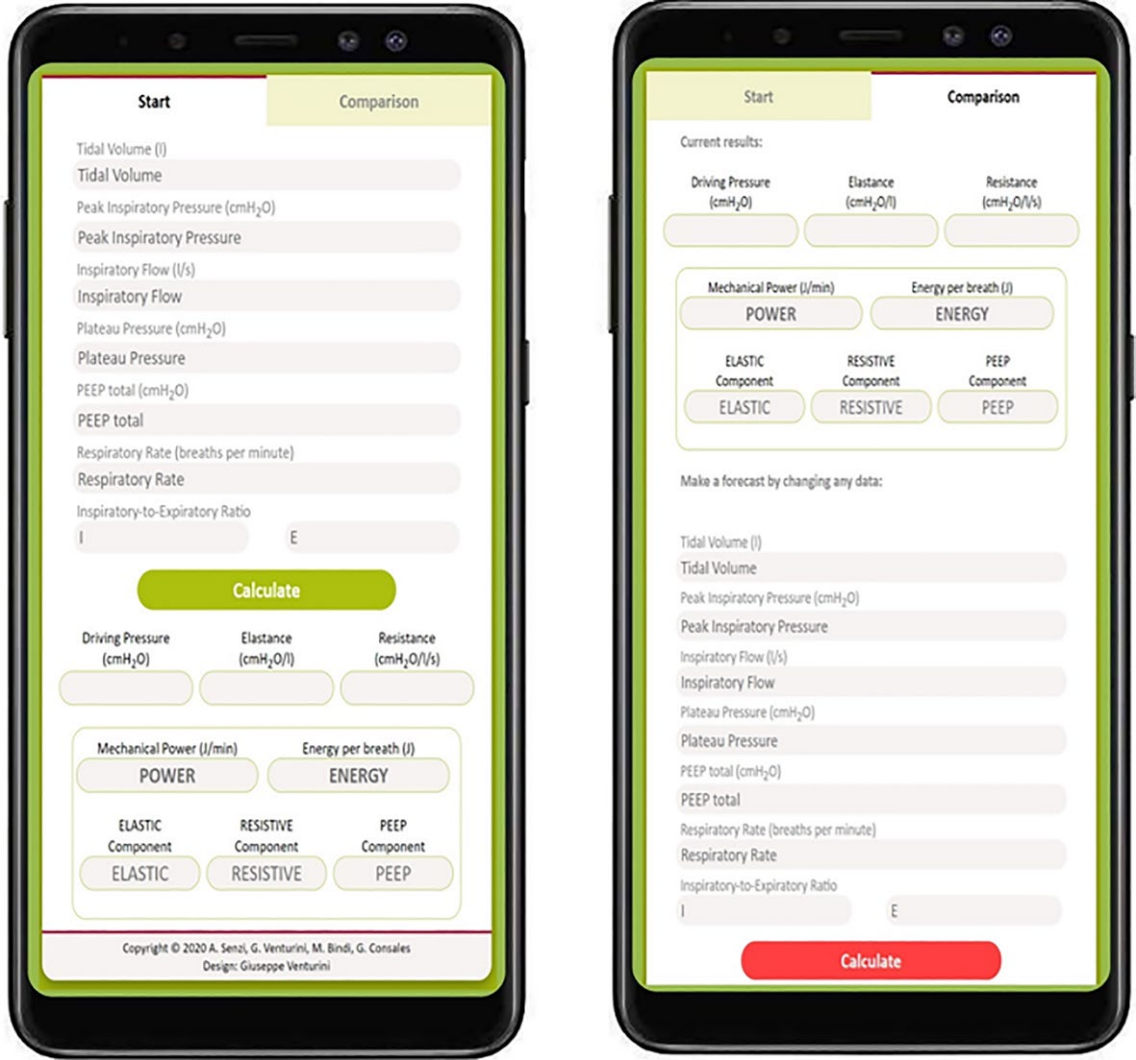
Main section of the “PowerApp” with the calculation tool for mechanical power and its relative components (elastic, resistive and PEEP component). The first section-tab “Start” allows to enter data easily available bedside from the ventilator; the output section firstly shows the calculated driving pressure (cmH_2_O), elastance (cmH_2_O/l) and inspiratory airway resistance (cmH_2_O/l/s), followed by mechanical power (J/min), energy per breath (J) and its relative components (%). The next section-tab “Comparison” initially displays the current results and allows the clinician to predict the variations in mechanical power, energy and its components by changing the ventilator setting or the physiological conditions

As shown in Fig. 1, the main page of the “PowerApp” allows to access the calculation tool, which is in turn divided in two section-tabs: in the first one (named “Start”) the user enters the measured variables, easily available at the bedside (i.e., peak and plateau pressure, PEEP, tidal volume, flow, respiratory rate, I:E ratio), to calculate the driving pressure, the elastance and the airway resistance in the output section, followed by the mechanical power, the energy per breath and the percentage contribution of each component. Mechanical power is calculated through the original formula by Gattinoni [1].

The next section-tab “Comparison” initially displays the current (uneditable) results and allows the clinician to change any of the former parameters predicting the variations in mechanical power, energy and its components according to new ventilator setting or different physiological conditions.

The second page allows to access the “Behind the app” part, with references and explications about the formula and the concept of mechanical power.

In conclusion, “PowerApp” allows to measure mechanical power at a glance, bedside, during mechanical ventilation setting and monitoring, without complex mathematical computation, making the power concept useful and feasible for everyday clinical practice.

## Data Availability

The material used and/or analyzed during the current study are available from the corresponding author on reasonable request. The mobile app created by the authors is freely available at https://mechpower.goodbarber.app.

## References

[1] Gattinoni L, Tonetti T, Cressoni M (2016). Ventilator-related causes of lung injury: the mechanical power. Intensive Care Med.

[2] Guérin C, Papazian L, Reignier J (2016). Effect of driving pressure on mortality in ARDS patients during lung protective mechanical ventilation in two randomized controlled trials. Crit Care.

[3] Coppola S, Caccioppola A, Froio S (2020). Effect of mechanical power on intensive care mortality in ARDS patients. Crit Care.

[4] Giosa L, Busana M, Pasticci I (2019). Mechanical power at a glance: a simple surrogate for volume-controlled ventilation. Intensive Care Med Exp.

[5] Silva PL, Ball L, Rocco PRM, Pelosi P (2019). Power to mechanical power to minimize ventilator-induced lung injury?. Intensive Care Med Exp.

